# Conservation Genomics for Threatened New Zealand *Gentianella calcis* (Gentianaceae) and Implications for Vulnerable Limestone Ecosystems

**DOI:** 10.1002/ece3.71596

**Published:** 2025-06-17

**Authors:** Robb W. Eastman‐Densem, David S. Glenny, Peter B. Heenan, Jana R. Wold, Pieter B. Pelser

**Affiliations:** ^1^ School of Biological Sciences University of Canterbury Christchurch New Zealand; ^2^ Allan Herbarium Manaaki Whenua − Landcare Research Lincoln New Zealand

**Keywords:** genotyping‐by‐sequencing, *Gentianella*, limestone plants, naturally insular ecosystems, paralogy

## Abstract

In New Zealand, limestone habitats are a naturally insular ecosystem, and obligate limestone taxa are extremely vulnerable to habitat degradation and destruction. Many New Zealand endemic vascular plants obligate to limestone habitats are in urgent need of conservation management, but often there is a lack of knowledge to inform such actions. We used genotyping‐by‐sequencing to explore patterns of genetic diversity and connectivity in *Gentianella calcis*, a threatened limestone gentian with four subspecies that are endemic to the eastern part of the South Island. We show that these subspecies and their populations are strongly genetically differentiated and have limited genetic connectivity. Two main genetic groups were identified. One of these comprises *G. calcis* subsp. *waipara* (North Canterbury) and the other consists of *G. calcis* subsp. *calcis*, *G. calcis* subsp. *manahune* and *G. calcis* subsp. *taiko* (South Canterbury and North Otago). Although evidence of Isolation‐By‐Distance suggests that the strong population differentiation is a result of restricted gene flow among populations, potential signatures of local adaptation were also seen. Observed heterozygosity showed some variation between sampled populations, with this possibly reflecting differences in population histories as well as the effects of paralogy in some SNPs. Overall, our data suggest that conservation of all extant populations is needed to effectively conserve genetic diversity in *G. calcis.* However, because of resourcing limitations, the conservation of some populations may need to be prioritised over that of others.

## Graphical abstract

Genotyping‐by‐sequencing revealed high infraspecific population structure and limited connectivity in the endangered *Gentianella calcis*, which is endemic to limestone ecosystems of the eastern South Island of New Zealand. These results suggest that conservation of all extant populations is needed to preserve current standing diversity. However, because of resourcing limitations, the conservation of some populations may need to be prioritised over that of others.

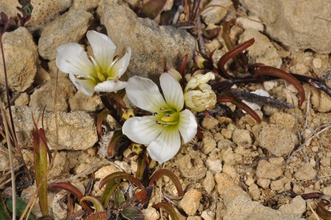

## 1 Introduction

Naturally insular specialist ecosystems represent important components of terrestrial plant biodiversity. They may support relict populations with unique diversity (Greimler and Dobeš 2000; Vogler and Reisch 2013) or hold the last remnants of native vegetation (de Vieira et al. 2015). Safeguarding these ecosystems is therefore important for preserving terrestrial biodiversity.

Molecular genetic studies have illustrated that plant species from naturally insular or specialist habitats often have high genetic structure (Bezemer et al. 2019; Khan et al. 2018; Mota et al. 2020). While this may be a result of the physical isolation of populations from one another (Gao et al. 2015; Mota et al. 2020), other aspects of these ecosystems, such as unique edaphic (soil) properties or chemistries (Folk and Freudenstein 2015; Nagasawa et al. 2020), can also drive population divergence. Such environmental differences among populations in combination with their isolation can result in genetic diversity with neutral (e.g., isolation) and non‐neutral (e.g., environmental) components, with these components potentially showing different genetic patterns across space (Orsini et al. 2013).

Species restricted to rare naturally insular ecosystems typically have small effective population sizes, which can make them particularly prone to losing genetic diversity. Considering that many rare naturally insular ecosystems are threatened by, e.g., quarrying, climate change, land conversion, habitat degradation and invasion of non‐indigenous weedy plants (Cartwright 2019; Clements et al. 2006; Molloy 1994; Willis et al. 1996), these characteristics make species of these ecosystems especially vulnerable. This may lead to a decline of the size and genetic diversity of populations of these species, potentially resulting in an ‘extinction vortex’ (Fagan and Holmes 2006; Gilpin and Soulé 1986).

In Aotearoa/New Zealand, calcareous (limestone) geology provides an example of naturally insular ecosystems of conservation concern (Holdaway et al. 2012). Much of the limestone in New Zealand, and particularly of eastern Te Waipounamu/South Island, occurs as small and scattered outcrops (see Heenan and Rogers 2019, Appendices 1–3). Prior to human settlement, limestone habitats likely would have been isolated islands of herbs, shrubs and grasses widely dispersed across lowland conifer‐hardwood forest (Heenan and Rogers 2019; McGlone 1989; Molloy 1994). The removal of forest and subsequent land changes (e.g., farming and quarrying) during human settlement significantly impacted limestone ecosystems (Holdaway et al. 2012; Molloy 1994). Indeed, in New Zealand over a quarter of vascular plant taxa that are obligate or strongly facultative to limestone habitats are currently assessed as having the highest conservation status of “Threatened, Nationally Critical” (Rogers et al. 2018), using the New Zealand Threat Classification System (Townsend et al. 2008).

*Gentianella* Moench (Gentianaceae) comprises approximately 300 herbaceous plant species from temperate or alpine areas throughout Eurasia, the Americas, Australia and New Zealand (von Hagen and Kadereit 2001), including several threatened or endangered taxa from limestone ecosystems (Greimler and Dobeš 2000; Oostermeijer et al. 2002; Pringle 2017). *Gentianella calcis* Glenny & Molloy (Glenny 2004) is endemic to limestone areas of the eastern South Island of New Zealand and is particularly threatened. Because it has few and small populations (de Lange et al. 2024; Frank 2023; Glenny 2004; Heenan and Rogers 2019), it is especially vulnerable to habitat degradation and destruction. Four *G. calcis* subspecies are currently recognised (Glenny 2004) and all are assessed as “Threatened, Nationally Critical” (de Lange et al. 2024; Rogers et al. 2018). This highlights the need for conservation management that uses patterns of genetic diversity and connectivity to identify conservation units (CUs), which are populations or groups of populations that are considered distinct for the purpose of conservation (Funk et al. 2012). Designating CUs is an important step in guiding conservation and management as they allow discrimination of different management areas, facilitating the application of policy, protection and monitoring (Coates et al. 2018; Funk et al. 2012). Additionally, this classification can communicate which populations could be mixed if translocations are considered, minimising the risk of outbreeding depression (Coates et al. 2018).

This study aimed to use single‐nucleotide polymorphism (SNP) markers obtained from genotyping‐by‐sequencing (GBS) to assess genetic diversity within and among subspecies and sampled populations of *G. calcis* and understand patterns of genetic structure, including neutral and adaptive diversity. We then sought to inform conservation efforts by using this information to designate conservation units and provide conservation management recommendations for *G. calcis*.

## 2 Materials and Methods

### 2.1 Study System and Specimen Sampling

**FIGURE 1:**
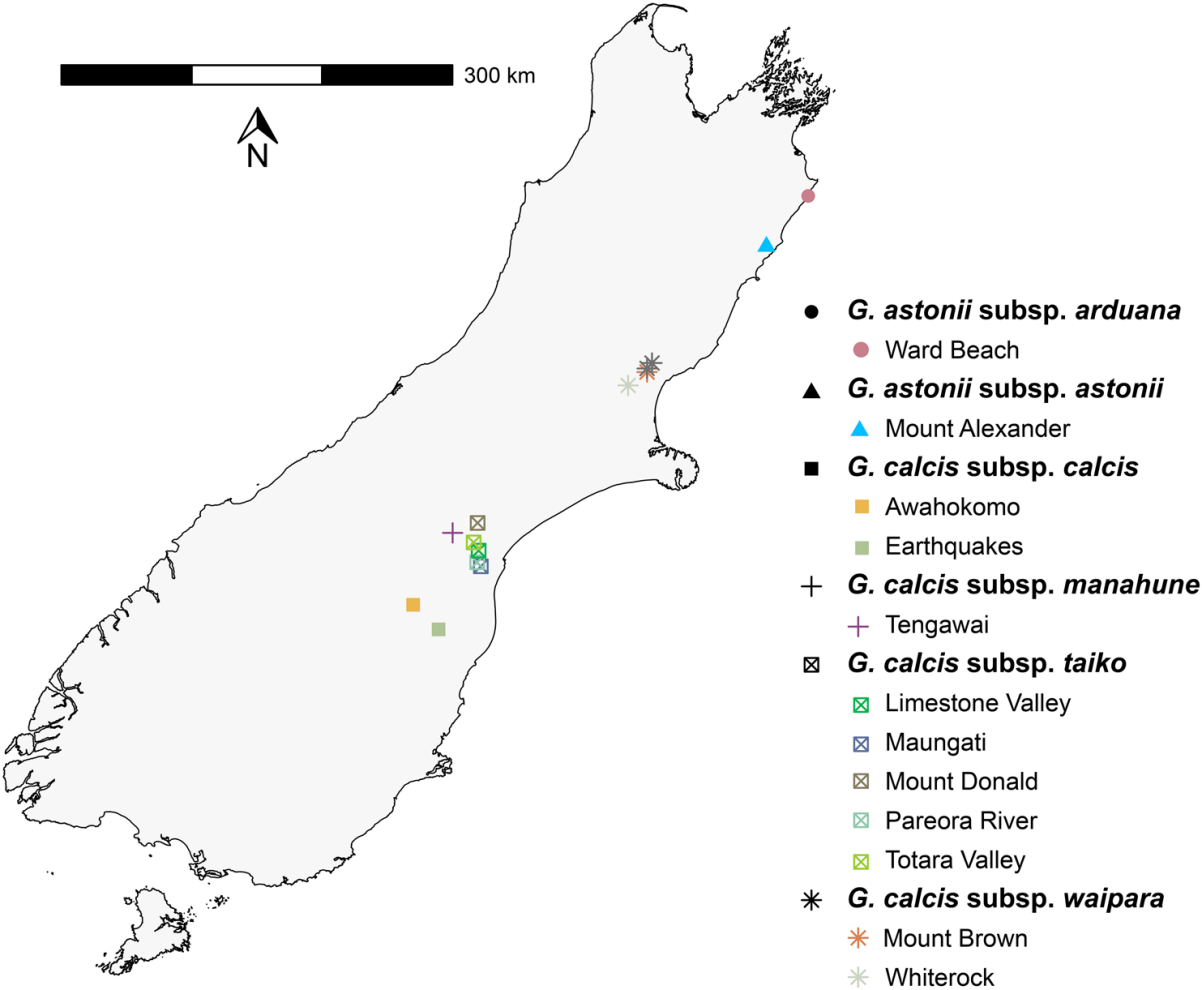
Map of the South and Stewart islands of New Zealand showing the location of each population of *G. calcis* and *G. astonii* sampled as part of this study (coloured shapes), as well as unsampled *G. calcis* populations (dark grey shapes).

*Gentianella calcis* comprises four subspecies: one in North Otago (*G. calcis* subsp. *calcis*), two in South Canterbury (*G. calcis* subsp. *taiko* Glenny & Molloy and *G. calcis* subsp. *manahune* Glenny & Molloy) and one in North Canterbury (*G. calcis* subsp. *waipara* Glenny & Molloy; Glenny 2004; Figure 1). Although no molecular phylogenetic studies have considered *G. calcis* to date, patterns of morphological similarities resulting from the most recent taxonomic revision of *Gentianella* in New Zealand (Glenny 2004) suggest that the *G. calcis* subspecies are closely related to another eastern South Island limestone endemic, *G. astonii* (Petrie) T.N.Ho & S.W.Liu, which has two subspecies (*G. astonii* subsp. *arduana* Glenny & Molloy and *G. astonii* subsp. *astonii*) that are both found in North Canterbury and Marlborough (Glenny 2004) and are assessed as “At Risk, Naturally Uncommon” by de Lange et al. (2024). We included representatives of *G. astonii* in our dataset as including less threatened closely related taxa in conservation genetics studies can provide useful context for interpreting patterns of genetic differentiation and diversity within the endangered species of interest (Llorens et al. 2018; Orel et al. 2024). A further reason to include *G. astonii* was to better elucidate its relationship to the subspecies of *G. calcis*, and in particular *G. calcis* subsp. *waipara* to which it is geographically close in North Canterbury (Glenny 2004).

Sampling was undertaken in November–December 2022 and February–March 2023 at 10 of the 12 known locations of *G. calcis* (Figure 1; Table 1). Aside from two populations of *G. calcis* subsp. *waipara*, this represented every known location of each subspecies (Figure 1). For *G. astonii*, specimens from two locations were selected to represent both subspecies (Figure 1). At each location, young‐looking green leaves, immature flowering stems and/or whole rosettes taken from plants were placed immediately onto silica gel in a resealable bag. Dry samples were stored in a dark cupboard at ambient room temperature until extraction < 3 months after collection. Voucher specimens were lodged at the University of Canterbury (CANU) and Allan (CHR) herbaria.

**TABLE 1:** Census sizes (number of plants), number of samples in datasets 2a and 3 (*n*) and genetic diversity metrics including number of private Single Nucleotide Polymorphisms (SNPs), SNP observed heterozygosity (*H*_o_), SNP expected heterozygosity (*H*_e_), SNP nucleotide diversity (*π*), inbreeding coefficient for SNP sites (*F*_IS_), autosomal observed heterozygosity (aH_o_), autosomal expected heterozygosity (aH_e_) and percentage of polymorphic loci for each sampled population of *G. calcis* and *G. astonii* in this study.

Taxon	Population	Census size	*n* dataset 2a	*n* dataset 3b	Number of private SNPs	SNP *H*_o_ (SE)	SNP *H*_e_ (SE)	SNP *π* (SE)	SNP *F*_IS_ (SE)	aHo (SE)	aHe (SE)	Percentage of polymorphic loci
*G. astonii* subsp. *arduana*	Ward Beach	Locally abundant	13	10	19	0.154 (0.013)	0.096 (0.008)	0.101 (0.008)	−0.104 (0.057)	0.00033 (0.00003)	0.00021 (0.00002)	0.046
*G. astonii* subsp. *astonii*	Mount Alexander	Locally abundant	10	5	6	0.117 (0.011)	0.078 (0.007)	0.084 (0.007)	−0.063 (0.054)	0.00025 (0.00003)	0.00017 (0.00002)	0.043
*G. calcis* subsp. *calcis*	Awahokomo	1918	14	6	1	0.094 (0.010)	0.069 (0.006)	0.073 (0.007)	−0.042 (0.067)	0.0002 (0.00002)	0.00015 (0.00001)	0.041
	Earthquakes	~100	13	11	0	0.093 (0.009)	0.071 (0.006)	0.074 (0.007)	−0.035 (0.060)	0.0002 (0.00002)	0.00015 (0.00001)	0.042
*G. calcis* subsp. *manahune*	Tengawai	322	25	16	36	0.114 (0.010)	0.086 (0.007)	0.088 (0.007)	−0.044 (0.090)	0.00024 (0.00002)	0.00018 (0.00002)	0.059
*G. calcis* subsp. *taiko*	Limestone Valley	> 500	5	3	0	0.128 (0.012)	0.078 (0.007)	0.091 (0.008)	−0.062 (0.034)	0.00027 (0.00003)	0.00017 (0.00002)	0.037
	Maungati	27	4	3	0	0.109 (0.011)	0.066 (0.006)	0.08 (0.008)	−0.049 (0.027)	0.00023 (0.00003)	0.00014 (0.00001)	0.034
	Mount Donald	125	12	8	4	0.123 (0.012)	0.078 (0.007)	0.083 (0.007)	−0.078 (0.054)	0.00026 (0.00003)	0.00017 (0.00002)	0.041
	Pareora River	22	3	3	0	0.115 (0.012)	0.071 (0.007)	0.091 (0.009)	−0.038 (0.021)	0.00024 (0.00003)	0.00015 (0.00002)	0.034
	Totara Valley	> 1000	16	6	2	0.117 (0.010)	0.087 (0.007)	0.091 (0.007)	−0.05 (0.074)	0.00025 (0.00002)	0.00019 (0.00002)	0.056
*G. calcis* subsp. *waipara*	Mount Brown	> 300	18	5	6	0.215 (0.014)	0.147 (0.009)	0.152 (0.009)	−0.127 (0.078)	0.00046 (0.00003)	0.00031 (0.00002)	0.078
	Whiterock	> 100	16	9	9	0.257 (0.016)	0.158 (0.009)	0.164 (0.009)	−0.177 (0.070)	0.00055 (0.00004)	0.00034 (0.00002)	0.077

*Note:* Census sizes reflect the most recent available estimate and use data from Frank (2023; *G. calcis* subsp. *taiko* and *G. calcis* subsp. *manahune*); Miller (2023; Awahokomo *G. calcis* subsp. *calcis*); D. Kimber, pers. comm. (2023; Whiterock *G. calcis* subsp. *waipara*); A. Miller (unpublished data; Mount Brown *G. calcis* subsp. *waipara*); Milliken et al. (2024; Earthquakes *G. calcis* subsp. *calcis*) and personal observations.

Abbreviation: SE, standard error.

In some locations, only a small number of *G. calcis* plants could be collected. In addition, some samples did not yield enough DNA of a suitable quality for sequencing. We therefore used tissue from wild‐sourced cultivated plants and of known provenance that were grown at Manaaki Whenua—Landcare Research, the Department of Conservation (DOC) field station nursery at Oamaru and a private residence instead of, or in addition to, field‐collected samples for Tengawai (one sample), Totara Valley (three samples) and Earthquakes (four samples). Although the absolute number of sampled *G. calcis* locations is low (i.e., two locations or fewer) for *G. calcis* subsp. *calcis, G. calcis* subsp. *manahune* and *G. calcis* subsp. *waipara,* samples were obtained from all but two of the known locations of this species.

### 2.2 Molecular Genetic Methods

DNA was extracted from 174 *G. calcis* and *G. astonii* samples using a DNeasy plant mini kit (Qiagen, Valencia, CA, USA). In addition, 14 replicate samples (10 technical replicates and four biological replicates) were included in the analyses as recommended by Mastretta‐Yanes et al. (2015). DNA quantification and assessment of purity was performed using the spectrophotometer on a DeNovix DS‐11 FX+ (DeNovix, Wilmington, Delaware) and a Qubit 2.0 fluorometer (Life Technologies, Carlsbad, California). Additionally, all samples were visually assessed for degradation on a 1% agarose gel. Samples with DNA concentrations between 25 and 100 ng/μL that appeared to be relatively pure and showed little visual degradation, were selected for sequencing. GBS data were generated following Elshire et al. (2011). 100 ng of genomic DNA and 3.6 ng of total adapters were used, genomic DNA was restricted with *ApeKI* and the library was amplified with 18 PCR cycles. 150 bp paired‐end sequences were generated using the Illumina NovaSeq platform (Illumina Inc., San Diego, CA, USA).

Library preparation and sample demultiplexing were performed by The Elshire Group Limited (Palmerston North, New Zealand). Sequencing produced 714 million read pairs, with an average of 3.7 million read pairs per sample. Raw sequence reads were demultiplexed using *Axe‐Demux* v0.3.3 (Murray and Borevitz 2018) with the *‐m* flag set to 0, and readthrough adapters removed using *TrimGalore* v0.6.7 (Krueger et al. 2021; Martin 2011). We used *FastQC* v0.12.1 (Andrews 2010) to determine read lengths, G/C content, and record other sequence information, and aggregated reports with *MultiQC* v1.14 (Ewels et al. 2016). Due to the presence of some relatively short reads, *TrimGalore* v0.6.7 was used to select only those with a length of at least 100 bp (*‐‐length* 100), with other parameters kept as default. As preliminary investigation of a dataset containing all samples indicated that samples with fewer reads were disproportionately contributing to missingness (results not shown), we retained only samples with at least 50% of the mean number of reads of at least 100 bp for further analysis to reduce missingness in the final dataset. A total of 160 samples met these criteria. *De novo* SNP discovery used the *Stacks* v2.64.0 pipeline (Catchen et al. 2011, 2013; Rivera‐Colón and Catchen 2022; Rochette et al. 2019).

As per Paris et al. (2017), we performed initial parameter optimisation trials in *Stacks*, using 12 samples (chosen to maximise location/taxon representation), with the *M* parameter in *ustacks* initially varied between 1 and 6. For *M* 1, the *n* parameter in *cstacks* was set at 1 and 2, while for *M* 2–6, values of *n* = *M* and *n* = *M* +/−1 were used. For all trials, the *m* parameter in *ustacks* was kept at the default value (i.e., *m* 3). This yielded a total of 17 different parameter combinations. Although Paris et al. (2017) suggested that the optimal parameter settings are those yielding the highest number of polymorphic loci in 80% of individuals (*−R* 80), the number of loci continued to increase up to *M* 6 in our trials (Table S1). Even for taxa with divergent or diverse populations, Paris et al. (2017) suggested that *M* values around 3 may be sufficient to capture polymorphism without increasing error, with higher *M* values also not recommended for organisms with repetitive or polyploid genomes (Paris et al. 2017), such as members of the Gentianaceae (Chen et al. 2021; Zhou et al., 2022). Thus, as a second optimisation method, we employed the approach of Mastretta‐Yanes et al. (2015) who considered that the best parameter combinations are those that yield the highest number of polymorphic loci while also reducing SNP error (defined as the number of differences in SNPs between sample replicate pairs divided by the total number of SNPs). To do this for our dataset, any replicate pairs available for the 12 optimisation samples (i.e., seven replicates) were added to the parameter trials, resulting in a total of 19 optimisation trial samples. Trials proceeded as before except that *M* was only varied from 1 to 4 (Table S1). Variant Call Format (VCF) files for each parameter combination were imported and manipulated in RStudio 2024.4.0.735 (Posit Team 2024) running R 4.2.2 (R Core Team 2022) using ‘read.vcfR’ and ‘vcfR2genlight’ from *vcfR* v1.13.0 (Knaus and Grünwald 2016, 2017). For each of the seven sample‐replicate pairs, SNP error was calculated and used alongside the number of polymorphic loci present in 80% of all samples. This showed that although higher *M* and *n* values provided a greater number of loci, they also increased SNP error (Table S1). Although parameter combinations *M* 1 *n* 1 and *M* 2 *n* 1 provided similar SNP error values as *M* 2 *n* 2, these yielded fewer loci, which indicated that these combinations were less optimal. As a result, we selected *M* 2, *n* 2 and *m* 3 as the parameter combination for final SNP discovery as it appeared to maximise the number of RAD loci while also reducing SNP error.

Following parameter optimisation, *Stacks* v2.64.0 was run for all 160 samples that passed initial quality control. To reduce computing time in *cstacks*, the locus catalogue was built using the three unique samples (i.e., not including replicates) from each sampling location with the greatest number of reads of at least 100 bp. On average 42.98% of sample reads (standard deviation 18.10%) mapped to catalogue loci in *cstacks*. After *gstacks*, per sample depth ranged between 5.9 and 12.8 (mean 8.5, standard deviation 1.4). Only 0.2% of loci could not be assembled into a paired‐end contig, with the remaining 99.8% forming contigs of an average of 157.7 bp in length. In total, *Stacks* genotyped 5,402,104 loci.

Once the main pipeline was completed, we first used *HDplot* (McKinney et al. 2017) to detect SNPs of putatively paralogous origins. This is important, because paralogs are a major source of error in *de novo* RAD‐seq datasets (e.g., Mastretta‐Yanes et al. 2015; Verdu et al. 2016). To perform the *HDplot* analyses, we exported SNPs with a minimum minor allele frequency (MAF) of 0.05 from RAD loci found in 80% of all samples (*−R* 80) to a VCF file using *populations*. Replicate samples were not included. As *HDplot* performs better with filtered data, we used these preliminary filters and thresholds to remove potentially spurious SNPs and reduce missingness. This created dataset 1, containing 149 samples and 1655 SNPs (Table 2), which was used to detect SNPs of putative paralogous origins using the *HDplot* functions in R (McKinney et al. 2017). Any SNP with the expected proportion of heterozygous individuals (*H*) greater than 0.5 and a “read‐ratio deviation” (*D*) less than −4 or greater than 4 was assumed to be paralogous, and any RAD locus containing at least one paralogous SNP considered a paralog. *HDplot* identified 291 putatively paralogous loci, and these were excluded from the dataset by rerunning *populations* using a blacklist and the following filters: one random SNP per RAD locus, maximum observed heterozygosity (*H*_o_) of 0.75, a minimum MAF of 0.05, RAD locus found in at least 80% of samples (−*R* 80). This created dataset 2a, containing 149 samples and 609 SNPs (Table 2), which was used for population genetic analyses. Although filtering trials using different minor allele and missingness cutoffs resulted in a greater number of SNPs and Principal Component Analyses (PCA) of these data sets showed highly similar patterns of genetic structure (Figures S1 and S2), their first four axes explained less of the variation observed, potentially indicating more random variation or error.

**TABLE 2:** Key filtering parameters and purpose of each major dataset constructed for this study.

Dataset		Replicates present	Maximum observed heterozygosity	Paralogs removed	Random SNP per RAD locus	Number of SNPs	*n*	*n* (replicates)	Purpose pf dataset
1		No	0	No	No	1655	149	0	Used for paralog detection
2	a	No	0.75	Yes	Yes	609	149	0	Used for population genetic analyes
	b	Yes	0.75	Yes	No	803	160	11	Used to estimate SNP error in dataset 2a
3	a	No	0	No	No	9403	85	0	Used to detect paralogs in dataset 3
	b	No	0.75	Yes	Yes	3280	85	0	Used for outlier analyses

*Note:* All datasets have a minimum minor allele frequency (MAF) of 0.05 and contain only samples with 50% or more of the mean number of reads with lengths greater than 100 bp. Datasets 3a and 3b additionally only contain samples with less than 50% of the mean number of SNPs missing from dataset 2a.

Abbreviation: *n*, number of samples.

To estimate SNP error, a second version of dataset 2 was created (Table 2). This contained all samples including replicates (i.e., 160 samples). To ensure results from analyses of dataset 2b could be compared with those of dataset 2a, only SNP loci in dataset 2a (i.e., those that passed filtering) and the same settings of *populations* were used before SNP error was calculated as previously described. SNP error was calculated as 0.0357 (3.57% of SNPs being different between sample‐replicate pairs) in dataset 2b.

To determine the relative contribution of putatively adaptive and putatively neutral processes to patterns of genetic diversity in *G. calcis* and *G. astonii*, we created a separate dataset for outlier detection. As outliers usually represent only a small subset of SNPs (Nielsen et al. 2020), to increase the number of SNPs in the dataset, we identified samples from dataset 2a with more than the mean number of SNPs missing using *vcftools* v0.1.13 (Danecek et al. 2011). We then created a new dataset (3a; Table 2) by excluding these samples and filtering for SNPs found in a minimum of 80% of all samples and minimum MAF of 0.05. Due to the increased number of SNPs in this dataset (9403), we used *HDplot* with the same settings as before to detect any paralogs not found previously and then *populations* and the same approach for filtering dataset 2a to create dataset 3b (Table 2). This increased the number of variant sites present across all samples compared to dataset 2a at the expense of reduced sample sizes for some populations. Dataset 3b contained 85 samples and 3280 SNPs and was used for outlier detection and analysis.

### 2.3 Genetic Structure

Unless specified, population structure analyses of dataset 2a were performed in RStudio 2024.4.0.735 (Posit Team 2024) using R 4.2.2 (R Core Team 2022). VCF files were imported and manipulated using ‘read.vcfR’ and ‘vcfR2genlight’ from *vcfR* v1.13.0 (Knaus and Grünwald 2016, 2017).

Principal Component Analysis (PCA) was performed using ‘glPCA’ with scaling from *adegenet* v2.1.10 (Jombart 2008; Jombart and Ahmed 2011). Five PCA axes were retained. Eigenvalues and PCA scores were plotted using *ggplot2* v3.4.4 (Wickham 2016) and 95% confidence ellipses were added to sampling locations.

To provide insight into hierarchical patterns of population structure and admixture, we used *STRUCTURE* (Pritchard et al. 2000). A compatible file was created by *PGDSpider* v2.1.1.5 (Lischer and Excoffier 2012) and *STRUCTURE* v2.3.4 (Pritchard et al. 2000) was run through *Structure_threader* v1.3.10 (Pina‐Martins et al. 2017) using the admixture model. *STRUCTURE* was run for *K* 2–12 (the number of sampling locations) for 20 iterations, each with 200,000 repetitions, with the first 100,000 discarded as burn‐in. The Evanno method (Evanno et al. 2005) was used as part of the wrapper in *Structure_threader* to select the optimal value for *K*. Visualisation of membership coefficients used *DISTRUCT* (Rosenberg 2004) and *CLUMPAK* (Kopelman et al. 2015) through the *StructureSelector* web tool (Li and Liu 2018).

A neighbour network graph of pairwise Nei's genetic distances (Nei 1972) between individuals was generated with *SplitsTree4* v4.19.0 (Huson 1998). These distances were calculated using ‘stamppNeisD’ from *StAMMP* v1.6.3 (Pembleton et al. 2013).

As the results of the PCA, *STRUCTURE* and *SplitsTree* analyses indicated that *G. calcis* and *G. astonii* are highly structured into populations corresponding to sampling locations, these were used for population assignment in a subsequent Analysis of Molecular Variance (AMOVA) to quantify the degree of population structure. This was performed at the population level using ‘poppr.amova’ in *ade4* v.1.7‐20 (Dray and Dufour 2007). Significance of population differentiation was tested using ‘randtest’ with 10,000 repetitions.

Pairwise *F*_ST_ values (Wright 1949; Weir and Cockerham 1984) among populations were calculated with ‘stammpfst’ from *StAMMP* using 50,000 bootstraps and a 95% confidence interval to provide estimates of pairwise population differentiation. A Bonferroni correction for multiple comparisons was applied with ‘p.adjust’. Additionally, we calculated private SNPs for all populations using the *populations* component of *Stacks* 2.64.0 (Catchen et al. 2011, 2013; Rivera‐Colón and Catchen 2022).

To determine if patterns of genetic structure can be explained by Isolation‐By‐Distance (IBD), the sampling coordinates of the 149 samples in dataset 2a were used to perform a Mantel test running for 9999 permutations with ‘gl.ibd’ in *dartR* v2.9.7 (Gruber et al. 2018; Mijangos et al. 2022) using Euclidean distance as the genetic distance between samples and Euclidean distance as the distance metric for geographic distance.

### 2.4 Genetic Diversity

Using *populations* in *Stacks* 2.64.0 with dataset 2a, we calculated population‐level diversity statistics including expected (*H*_e_) and observed heterozygosity (*H*_o_), nucleotide diversity (*π*) and inbreeding coefficients (*F*_IS_) from variant (SNP) sites only. Additionally, we calculated autosomal expected (aH_e_) and observed heterozygosity (aH_o_) and percentage of polymorphic sites using both variant and monomorphic sites as per Schmidt et al. (2021).

### 2.5 Outlier Analysis

To determine patterns of genetic structure shaped by local selection, we used *pcadapt* v4.4.3 for an outlier analysis (Luu et al. 2017). This approach aims to identify non‐neutral SNPs. We chose *pcadapt* for this purpose, because it is more tolerant of missing data and different types of population structure than other approaches (Luu et al. 2017). The ‘read.pcadapt’ function was used to import the VCF file of dataset 3b, and the proportion of variance explained by the first 20 principal components was initially investigated using the function ‘pcadapt’. We used Cattell's graphical rule to determine the appropriate number of PC axes for ‘pcadapt’ (Luu et al. 2017) which suggested the retention of 10 principal components. A Bonferroni correction was applied to the *p*‐value of each SNP using ‘*p*.adjust’, and all SNPs with an alpha below 0.01 were considered outliers. 439 out of 3280 SNPs were identified as outliers, and separate datasets for these and the remaining 2841 (neutral) SNPs were created, and a PCA was undertaken for each dataset following the method described previously. To help understand which populations could be grouped into CUs, additionally, we calculated pairwise *F*_ST_ using neutral SNPs for each population (as suggested by Funk et al. 2012) and tested their significance using the previously described method.

## 3 Results

### 3.1 Population Structure

**FIGURE 2:**
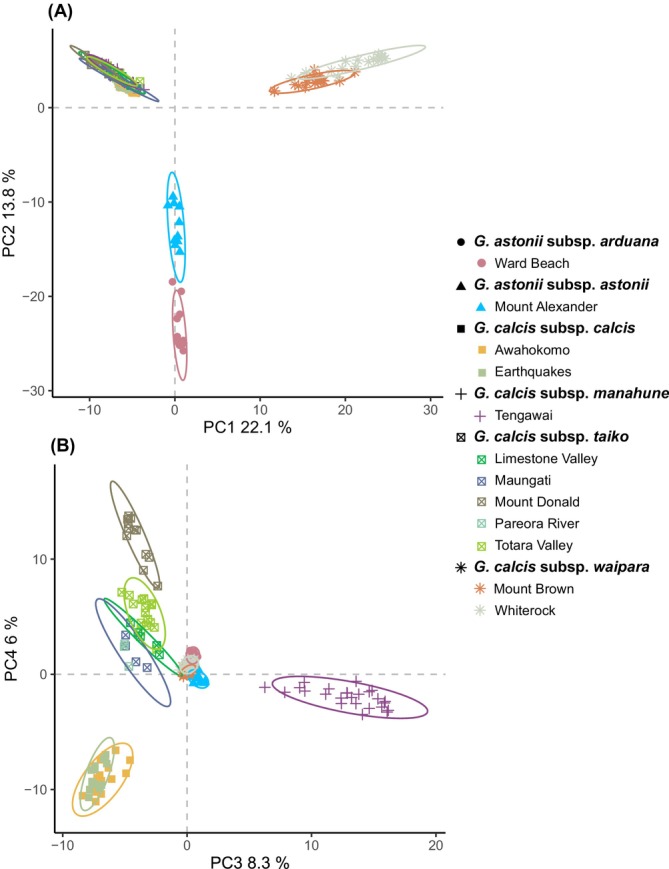
Principal Component Analysis (PCA) of dataset 2a showing first and second axes (A) and third and fourth axes (B) with subspecies and population of each sample indicated.

**FIGURE 3:**
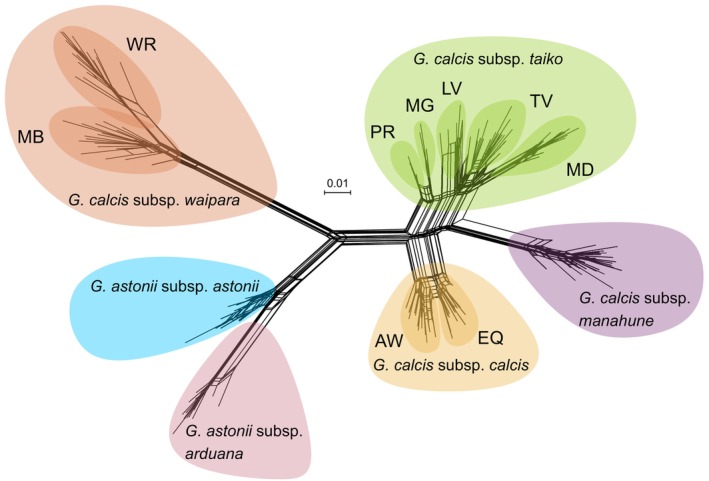
Neighbour Network Graph produced by *Splitstree* using pairwise Nei's genetic distances between all samples in dataset 2a with subspecies and sampling locations highlighted. Sampling locations are as follows: WB: Ward Beach; MA: Mount Alexander; AW: Awahokomo; EQ: Earthquakes; TG: Tengawai; LV: Limestone Valley; MG: Maungati; MD: Mount Donald; PR: Pareora River; TV: Totara Valley; MB: Mount Brown; WR: Whiterock.

**FIGURE 4:**
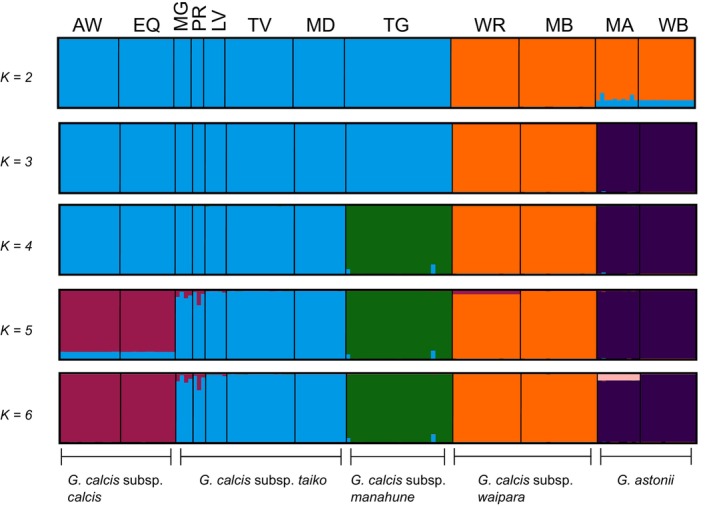
Hierarchical clustering plots of dataset 2a produced by *STRUCTURE* and visualised using *DISTRUCT* through *CLUMPAK* for *K* = 1–6. Sample location abbreviations are as follows: WB: Ward Beach; MA: Mount Alexander; AW: Awahokomo; EQ: Earthquakes; TG: Tengawai; LV: Limestone Valley; MG: Maungati; MD: Mount Donald; PR: Pareora River; TV: Totara Valley; MB: Mount Brown; WR: Whiterock.

The first two axes of the PCA of dataset 2a explained a total of 35.9% of the variation (Figure 2A). The PCA plot as well as the neighbour‐net graph from *SplitsTree* (Figure 3) showed three main groups: (1) *G. calcis* subsp. *calcis*, *G. calcis* subsp. *taiko* and *G. calcis* subsp. *manahune* (here referred to as the South Canterbury group), (2) *G. calcis* subsp. *waipara* and (3) *G. astonii*. When the third and fourth axes were considered, structuring by subspecies and substructuring by population was visible in the South Canterbury group. The Evanno method for inferring Δ*K* (Evanno et al. 2005) indicated that *K* = 2 was the optimal value for *K* (Figure S3). At this K value, *STRUCTURE* (Figure 4) divided the taxa into two groups, with one consisting of the South Canterbury group, while the other containing *G. calcis* subsp. *waipara* and *G. astonii*. Some potential assignment to the South Canterbury group was seen in *G. astonii*, however this disappeared at *K* = 3. At this value, the *G. astonii* samples formed a separate group, with the two remaining groups consisting of *G. calcis* subsp. *waipara* and the South Canterbury group. Higher *K* values generally showed a pattern of hierarchically structured groupings mostly corresponding to subspecies and/or sampled populations. Little admixture between populations was observed, except between the two sampled *G. calcis* subsp. *calcis* populations and some *G. calcis* subsp. *taiko* populations, with admixture seen at *K* ≥ 5 (Figure 4; *K* = 5, 6).

AMOVA partitioned approximately 75% of the variation in dataset 2a among populations (Table S2), which showed statistically significant differentiation (*p* < 0.05). Pairwise *F*_ST_ values between all *G. calcis* and *G. astonii* populations ranged between 0.133 and 0.670 (Table 3; lower triangle). All values were significant after a Bonferroni correction.

**TABLE 3:** Pairwise *F*_ST_ values (Wright 1949; Weir and Cockerham 1984) among populations using only neutral SNPs (dataset 3b; upper triangle) and all SNPs (dataset 2a; lower triangle). All values are significant after a Bonferroni correction for multiple comparisons.

	Tengawai	Awahokomo	Totara Valley	Earthquakes	Maungati	Mount Donald	Pareora River	Mount Alexander	Mount Brown	Limestone Valley	Ward Beach	Whiterock
Tengawai		0.463	0.417	0.490	0.423	0.473	0.437	0.556	0.587	0.412	0.643	0.631
Awahokomo	0.495		0.428	0.166	0.383	0.505	0.421	0.581	0.588	0.432	0.633	0.622
Totara Valley	0.420	0.372		0.428	0.326	0.332	0.327	0.597	0.592	0.335	0.644	0.631
Earthquakes	0.502	0.155	0.378		0.369	0.497	0.398	0.556	0.584	0.416	0.639	0.625
Maungati	0.457	0.388	0.257	0.338		0.453	0.229	0.623	0.590	0.322	0.633	0.624
Mount Donald	0.496	0.476	0.311	0.483	0.392		0.462	0.638	0.638	0.473	0.675	0.666
Pareora River	0.465	0.406	0.259	0.350	0.133	0.429		0.640	0.601	0.344	0.641	0.633
Mount Alexander	0.560	0.566	0.549	0.550	0.538	0.609	0.553		0.534	0.635	0.475	0.574
Mount Brown	0.550	0.508	0.509	0.492	0.437	0.552	0.446	0.459		0.598	0.600	0.214
Limestone Valley	0.454	0.415	0.255	0.406	0.323	0.403	0.308	0.573	0.480		0.642	0.631
Ward Beach	0.606	0.589	0.581	0.575	0.553	0.620	0.565	0.360	0.513	0.584		0.639
Whiterock	0.578	0.541	0.540	0.526	0.475	0.577	0.479	0.502	0.154	0.507	0.538	

A significant effect of IBD was observed (*p* < 0.05). Overall, geographic distance explained 49.9% of the variation in pairwise Euclidean distances between individual genotypes (Figure S4).

The number of private SNPs per population (Table 1) ranged from 0 to 36, with both *G. calcis* subsp. *manahune* and *G. astonii* subsp. *arduana* populations having more than 15 private SNPs. Conversely, the three southernmost *G. calcis* subsp. *taiko* populations (Limestone Valley, Pareora River and Maungati) and the Earthquakes population of *G. calcis* subsp. *calcis* had none.

### 3.2 Genetic Diversity

SNP observed heterozygosity and nucleotide diversity were highest in both populations of *G. calcis* subsp. *waipara* (*H*_o_: 0.215–0.257; *π*: 0.152–0.164; Table 1). *Gentianella astonii* subsp. *arduana* had moderately high observed heterozygosity (*H*_o_: 0.154) relative to other populations. The other populations, including South Canterbury *G. calcis* populations and *G. astonii* subsp. *astonii*, had similar estimates of observed heterozygosity (*H*_o_: 0.093–0.128). Nucleotide diversity was similar for these populations and *G. astonii* subsp. *arduana* (*π*: 0.073–0.101). For all populations, estimates of observed heterozygosity were higher than expected heterozygosity, leading to negative *F*_IS_ values (Table 1). Autosomal heterozygosity showed similar patterns to SNP heterozygosity, with autosomal observed heterozygosity especially high in the Whiterock population of *G. calcis* subsp. *waipara* (aH_o_: 0.0055). Percentages of polymorphic loci were highest in both *G. calcis* subsp. *waipara* populations (c. 0.08% polymorphic), with the remaining populations having similar, but slightly lower percentages (c. 0.03%–0.06% polymorphic).

### 3.3 Outlier Analysis

**FIGURE 5:**
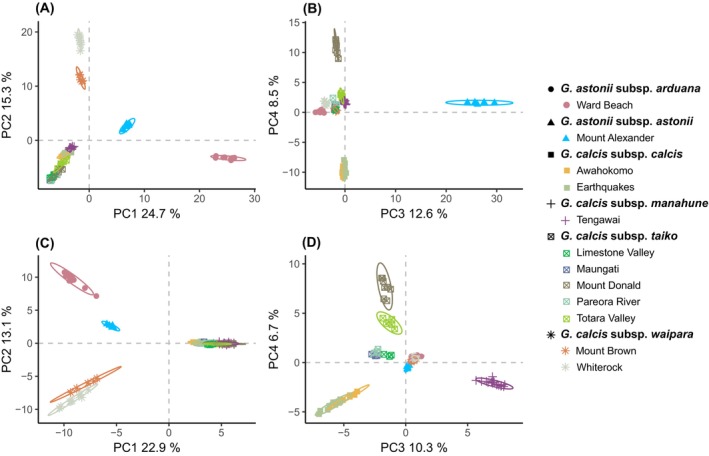
Principal Component Analysis (PCA) of dataset 3b showing first and second axes of outlier SNPs (A), third and fourth axes of outlier SNPs (B), first and second axes of neutral SNPs (C) and third and fourth axes of neutral SNPs (D) with subspecies and population of each sample indicated.

In the PCA of neutral SNPs (Figure 5), a pattern highly similar to that observed in the PCA using dataset 2a (i.e., all SNPs; Figure 2) was seen (Figure 5C,D). Although the same pattern was observed in the axis 1 versus 2 plot of the PCA results obtained from the putatively adaptive SNPs (Figure 5A), different relationships were displayed in the axis 3 versus 4 plot (Figure 5B). Here, *G. astonii* subsp. *astonii, G. calcis* subsp. *calcis* and the Mount Donald population of *G. calcis* subsp. *taiko* are more genetically differentiated from each other and the other populations than in the PCA of the neutral SNPs. Pairwise *F*_ST_ values between all populations using only neutral SNPs (Table 3; upper triangle) ranged between 0.166 and 0.675. All were significant after a Bonferroni correction.

## 4 Discussion

Conservation units provide a means to inform resource allocation and support conservation policy (Coates et al. 2018; Funk et al. 2012; Supple and Shapiro 2018). By identifying populations or groups of populations that show evolutionary novelty, or are genetically divergent, CUs can be used in efforts to protect infraspecific genetic diversity (Coates et al. 2018; Funk et al. 2012; Moritz et al. 2002). This may be particularly important for species with high infraspecific genetic structure, or historically isolated populations, as CUs can be used to inform translocations or prioritise protection of certain unique genetic diversity (Coates et al. 2018). Using our knowledge of the patterns of genetic diversity in *G. calcis*, we can designate CUs to assist with its conservation management.

### 4.1 Population Structure

Our results revealed that *G. calcis* has high infraspecific genetic structure and differentiation, with limited connectivity observed among subspecies. The most substantial genetic differentiation was observed between *G. calcis* subsp. *waipara* and the South Canterbury *G. calcis* populations (Figures 2, 3, 4), although the subspecies in the South Canterbury group were also genetically differentiated from each other. The high differentiation among the *G. calcis* subspecies suggest that reinvestigation of the taxonomic circumscription of *G. calcis* may be warranted. Although the infraspecific taxonomic delimitation of this species was based on limited material due to the lack of specimens at the time (Glenny 2004), greater interest in the group since this revision has resulted in a larger number of populations being discovered and available for study. This provides opportunities to revisit the taxonomic delimitation of *G. calcis* both at the species and infraspecific level.

*STRUCTURE* analyses suggested potential admixture between the most geographically proximate populations of *G. calcis* subsp. *taiko* and *G. calcis* subsp. *calcis*—which are separated by at least 50 km (Figures 1 and 4). Although *G. calcis* has not been recorded in the area between populations of these subspecies (Frank 2023; Glenny 2004), potentially suitable limestone habitat exists in the intervening landscape (Heenan and Rogers 2019). Therefore, admixture might have occurred because of migration via ‘stepping stone´ populations that are now either extinct or await discovery. Although some assignment to the South Canterbury group was also seen in *G. astonii* at *K* = 2 (Figure 4), this disappeared at higher values of *K* and was relatively equal across samples. This pattern may therefore represent an artefact of analysis or filtering, which can create a false signal of admixture (Linck and Battey 2019).

High genetic differentiation and limited connectivity were also observed at the population level, as evidenced by statistically significant pairwise *F*_ST_ values between all populations, the results of the AMOVA and the presence of private SNPs in many populations. PCA and *Splitstree* analyses provided similar patterns of genetic structure. IBD appeared to be a significant factor (*p* < 0.05) in explaining the genetic structure patterns in our data, accounting for nearly 50% of the variation seen (Figure S2). This was particularly evident for *G. calcis* subsp. *taiko*, whose populations were arranged in a north‐to‐south configuration in the PCA results (Figures 1 and 2B).

The high genetic differentiation among populations of *G. calcis* may have been facilitated by its naturally insular ecosystem, as limestone in the eastern South Island comprises small and isolated outcrops. The distances between these limestone areas may be sufficient to restrict gene flow among them, leading to differentiation via genetic drift. Indeed, even at relatively small geographic scales (i.e., tens of kilometres) significant population differentiation has been observed in other taxa endemic to naturally insular ecosystems (e,g., Llorens et al. 2018; Orel et al. 2024; Rusterholz et al. 2012; Silva et al. 2020). Other threatened plant taxa from eastern South Island limestone ecosystems such as *Australopyrum calcis* Connor & Molloy (Poaceae), *Geranium socolateum* Heenan & Molloy (Geraniaceae) and *Pimelea declivis* C.J.Burrows (Thymelaeaceae) have similar distribution patterns (Heenan and Rogers 2019) and are sympatric with *G. calcis* at many locations (pers. obs.), but there are currently no published genetic studies of these or other rare eastern South Island limestone endemics. It is therefore presently unknown if the patterns of genetic structure found for *G. calcis* are a general feature of the limestone‐endemic flora of the region. However, a population genetic study of *Gingidia enysii* (Kirk) J.W.Dawson (Apiaceae), a more widespread threatened species that is also partly sympatric with *G. calcis*, did not reveal a geographic signal in its genetic structure in eastern South Island limestone areas (Heenan et al. 2021). This suggests that habitat isolation may not always be a significant factor in creating population differentiation in this ecosystem. However, unlike *Gentianella calcis*, the distribution of *Gingidia enysii* on limestone is less disjunct as it occurs on many sites where *Gentianella* are not present (Heenan and Rogers 2019; Heenan et al. 2021). Its genetic structure might therefore have been impacted less by the insular nature of the eastern South Island limestone habitat, resulting in greater genetic connectivity among populations than seen in *G. calcis*. Additionally, *Gingidia* have wind‐dispersed seeds with winged projections and therefore may be more effective at long‐distance dispersal than *Gentianella*, which use censer dispersal (Thorsen et al. 2009) and have spherical seeds without ornamentation (Glenny 2004). For taxa from naturally insular ecosystems, limited seed dispersal ability has also previously been suggested as a key factor contributing to high population divergence (Llorens et al. 2018; Orel et al. 2024), indicating the importance of organismal traits in genetic responses to naturally insular ecosystems.

For both *G. calcis* and *G. astonii*, local adaptation could have contributed to population differentiation, as indicated by differences between genetic patterns obtained from putative adaptive vs. neutral SNPs (Figure 5). In the PCA plot of the third and fourth axes obtained from putative adaptive SNPs, greater differentiation was seen between the two *G. astonii* subspecies, with the Mt. Donald population of *G. calcis* subsp. *taiko* and *G. calcis* subsp. *calcis* also showing greater differentiation (Figure 5B). This suggests that both neutral and adaptive processes may have created population structure. Differences in habitat preference have been reported between the *G. calcis* subspecies, with *G. calcis* subsp. *taiko* preferring a moister habitat than the other taxa (Frank 2023; Glenny 2004). Additional differences have been noted between the Mt. Donald population of *G. calcis* subsp. *taiko* and the other populations of this taxon (Frank 2023). As well as occupying a more exposed habitat, the Mt. Donald population has morphological characteristics that are similar to those of *G. calcis* subsp. *manahune* (Frank 2023), and our results suggest that there may be an adaptive component to these observations.

Adaptive differentiation might be expected for *G. calcis* and *G. astonii* due to differences in local climatic conditions across their distribution. Climate has been shown to be important in explaining patterns of genetic diversity in widespread New Zealand plants such as *Kunzea* (Myrtaceae) (Heenan et al. 2024). Another environmental factor influencing *G. calcis* and *G. astonii* could be adaptation to specific substrates due to differing physical (e.g., particle size) and chemical (e.g., carbonate content) properties. The South Canterbury taxa are found on Otekaieke limestone, while *G. calcis* subsp. *waipara* favours the Mount Brown Formation and *G. astonii* subspecies occur on Amuri limestone (Mortimer and Strong 2014; Rogers et al. 2018). Variation in soil pH due to differing parent materials may have impacted the distribution of *Carmichaelia* (Fabaceae) species in Marlborough (Heenan 1996) and is therefore potentially important for *G. calcis* as well. Patterns suggesting fidelity to specific habitat soils or adaptation to local conditions are also seen in the limestone flora of New Zealand more broadly, with 78% of limestone plant taxa confined to single limestone types or regional groups (Rogers et al. 2018). As inhabitants of naturally fragmented ecosystems, such as *G. calcis*, may occupy microhabitats that are difficult to capture in traditional environmental models (Cartwright 2019), more specific research into differences in climate and substrates among populations is needed to determine what explains the greater genetic differentiation of the Mt. Donald population of *G. calcis* subsp. *taiko, G. calcis subsp. calcis*, as well as *G. astonii* subsp. *astonii* relative to the other populations, as visible in the PCA obtained from putative adaptive SNPs (Figure 5B). Overall, it appears that a combination of an insular habitat, organismal traits and environmental factors is likely responsible for the observed patterns of genetic structure within *G. calcis*. At the local scale, IBD caused by an insular habitat type appears to have been a major force restricting gene flow. At larger distances between populations, environmental differences may have further driven differentiation through adaptation to local conditions.

### 4.2 Genetic Diversity

Genetic diversity as measured by SNP nucleotide diversity, SNP heterozygosity and autosomal heterozygosity is similar among South Canterbury *G. calcis* populations and much higher in *G. calcis* subsp. *waipara* from North Canterbury. The more widespread *G. astonii* subsp. *arduana* and *G. astonii* subsp. *astonii* had only slightly higher or very similar estimates of genetic diversity compared with the South Canterbury *G. calcis* taxa. This was unexpected as smaller populations generally lose genetic diversity faster and have lower heterozygosity than larger populations, as inbreeding between close relatives is increased and the effects of genetic drift are stronger (Ellstrand and Elam 1993; Young et al. 1996). Both sampled populations of *G. calcis* subsp. *waipara*, however, had the highest genetic diversity estimates despite containing only c. 100–300 plants each (Table 1). Moreover, several *G. calcis* subsp. *taiko* populations with the smallest estimated population sizes at the time of sampling (Maungati: 27 plants; Pareora River: 22 plants) had estimates of genetic diversity that were very similar to the two largest populations of this subspecies (Totara Valley: > 1000 plants; Limestone Valley: > 500 plants), as well as *G. calcis* subsp. *manahune* (c. 300 plants) or *G. astonii* subsp. *astonii* (Table 1). Although heterozygosity is commonly considered when assessing the effects of fragmentation on genetic diversity (Aguilar et al. 2008), it may respond more slowly compared to other measures such as allelic richness and percentage of polymorphic loci (Aguilar et al. 2008; Honnay and Jacquemyn 2007). We note here that the Pareora River and Maungati populations had the lowest percentage of polymorphic loci in our dataset at 0.034% (Table 1). Although this is only about a 0.01% difference with other populations, it might suggest the importance of considering temporal aspects when interpreting observed patterns of genetic diversity. In New Zealand, human‐driven landscape changes only started becoming more intense 750 years ago with the widespread clearing of forest (McGlone 1989). As *G. calcis* favours open grassland or habitats occupied by other herbaceous species, this forest clearance may not have impacted this taxon as much as forest species. Indeed, many of the current threats to *G. calcis*, including agricultural intensification, grazing pressure, spread of non‐indigenous weedy plants and pest mammals, are threats that have only been present since European settlement (Cumberland 1941) in the last 200 years. Perhaps not enough time has passed for us to see the effects of fragmentation on genetic diversity in *G. calcis* populations. This could explain the relatively high genetic diversity in *G. calcis* subsp. *waipara*, as local populations may still contain relic diversity from when populations were perhaps more numerous or larger. Although the unequal sample sizes of populations may have contributed to bias in the estimates of SNP heterozygosity, our estimates of autosomal heterozygosity, which is shown to be less biased by sample size (Schmidt et al. 2021), also showed this pattern. Overall, this implicates other biotic and abiotic factors in *G. calcis* and *G. astonii* affecting genetic diversity such as demography, life history, mating system, selection or time since changes in land use as being more important determinants than population size (Ellegren and Galtier 2016).

### 4.3 Heterozygosity Excess, Paralogs and Sample Size Limitations

In all populations sampled, *H*_o_ was greater than *H*_e_, leading to negative *F*_IS_ values (Table 1). This heterozygosity excess was unexpected considering the small size, and therefore increased chance of inbreeding, of many *G. calcis* populations. Although negative *F*_IS_ is not uncommon in SNP‐based plant population genetic studies (e.g., Dalapicolla et al. 2021; Edgeloe et al. 2022; Lyu et al. 2020; Probowati et al. 2023; Ruiz Mondragon et al. 2022) and can indicate the existence of a recent bottleneck (Luikart and Cornuet 1998), the finding that all populations regardless of sample or census size exhibited this behaviour suggests that other explanations are more likely. Negative *F*_IS_ values can also indicate the presence of undetected substructure in our populations. However, inspection of pairwise kinship coefficients among individuals as calculated using *‐‐relatedness2* from *vcftools* v0.1.13 showed little evidence for substructure within populations (Figure S5). As heterozygote excess was observed in all populations, this hypothesis is also only poorly supported by our data. In considering excess heterozygosity in *Prunus avium* (L.) L. populations, Stoeckel et al. (2006) suggested that factors such as an outcrossing mating system, overdominance or clonality may create excess heterozygosity. As *G. calcis* and *G. astonii* are self‐compatible (Lord 2022; Milliken et al. 2024) and may only show limited clonality in the wild if at all (pers. obs.), only overdominance may be applicable. However, a cursory inspection of the differences in heterozygous and homozygous site frequency between putatively neutral and outlier SNPs (not shown) did not show patterns indicative of overdominance. A more likely explanation is the presence of paralogs inflating observed heterozygosity estimates. All *Gentianella* species in New Zealand are likely of polyploid origin (Glenny 2004; Weaver and Rüdenberg 1975), with other studies of the Gentianaceae suggesting members of this family may have highly repetitive or duplicated genomes (Chen et al. 2021; Zhou et al. 2022). Although we tried to detect and eliminate potentially paralogous SNPs, it is still possible that some were included in our dataset, increasing our heterozygosity estimates.

PCA analyses of datasets before (not shown) and after (Figure 2) *HDplot* functions were used to identify and remove paralogs, showing highly similar patterns of population structure. Furthermore, our estimate of SNP error (3.57%), although being slightly higher than the value reported for optimised *Stacks* parameters by Mastretta‐Yanes et al. (2015; i.e., 2.43%), is very similar to the value they report for near‐optimal *Stacks* parameters (3.21%). There are therefore no indications that any remaining paralogs significantly contributed to the genetic structure patterns that we obtained. Further, including paralogs mostly appears to decrease relative differentiation (Mastretta‐Yanes et al. 2014, 2015), reinforcing that it is unlikely that the strong patterns of population structure and divergence we observed are due to their inclusion.

Aside from the presence of paralogs in our data, another potential limitation of our study were the small sample sizes for several *G. calcis* populations (Table 1). Small sample sizes may lead to inaccurate estimates of population differentiation (*F*_ST_) and genetic diversity (Aguirre‐Liguori et al. 2020), particularly if sample sizes vary among populations (Sopniewski and Catullo 2024). In some circumstances, however, extremely small sampling sizes (e.g., 2–6 per population) have been shown to be sufficient for accurately estimating *F*_ST_ given many SNP markers (Nazareno et al. 2017; Willing et al. 2012). Although the relatively small number of SNPs in dataset 2a (609; Table 2) and uneven sampling sizes could have prevented accurate estimation of summary statistics for these populations, such constraints cannot be avoided in conservation genetic studies of threatened non‐model species with small population sizes and few extant populations. However, linear regressions to test for a relationship between sample size and observed heterozygosity, as well as sample size and *F*_IS_, were not statistically significant (Tables S3 and S4), suggesting that the relatively low sample sizes may not have had a substantial impact on our estimates of population differentiation and genetic diversity.

### 4.4 Conservation Implications for *Gentianella calcis*

Our results indicate that *G. calcis* populations are genetically divergent, with limited gene flow among them. It is therefore meaningful to designate infraspecific Conservation Units (CUs) such as Evolutionarily Significant Units (ESUs) and/or Management Units (MUs) to facilitate conservation and ensure protection of genetic diversity below the species level (Coates et al. 2018; Funk et al. 2012; Moritz et al. 2002).

As a first step in designating CUs, Funk et al. (2012) recommended using all loci (i.e., both neutral and adaptive) to delimit ESUs. Although ESUs are typically designated within subspecies (Coates et al. 2018), given that three of the four *G. calcis* subspecies have few extant populations and this limits the potential for infraspecific subdivision, each of its subspecies is best considered an ESU. MUs are typically defined as demographically independent populations (Funk et al. 2012; Palsbøll et al. 2007) and considered as such when their dispersal rates are < 10% (Palsbøll et al. 2007). Metrics of population differentiation such as *F*_ST_ can be used to estimate current rates of dispersal. However, this relies on knowledge of effective population size and requires assumptions of idealised populations (Palsbøll et al. 2007). Application of these metrics may therefore be difficult when insufficient population data exist (such as in *G. calcis*) and necessitate the use of non‐quantitative approaches (Forester et al. 2022). In our study of limestone gentians, we therefore used PCA and pairwise *F*_ST_ values from neutral SNPs to designate MUs. In our PCA plots (Figure 5C,D) we showed that the Tengawai (*G. calcis* subsp. *manahune*), Mount Donald and Totara Valley (*G. calcis* subsp. *taiko*) populations formed separate genetic clusters and are therefore likely demographically independent. This is further supported by the high pairwise *F*_ST_ values for these populations (i.e., 0.332–0.473; Table 3; upper triangle). Despite grouping together in the PCA plots, all other *G. calcis* populations also have high pairwise *F*_ST_ values, with Awahokomo and Earthquakes (*G. calcis* subsp. *calcis*) showing the lowest value of 0.166 (Table 3, upper triangle). This indicates that all sampled populations could be considered MUs within their respective subspecies and implies that each population may need to be conserved to preserve the standing genetic diversity of each subspecies.

This could be accomplished in the short to medium term through *ex situ* conservation of plants from every MU we have identified. For example, for small populations such as the Pareora River and Maungati populations of *G. calcis* subsp. *taiko*, plants grown from seed collected from these populations could be used to increase their population sizes, thereby reducing the risk of genetic diversity loss or inbreeding depression (Frankham et al. 2014). However, to protect populations long term, there is a significant need to address the direct causes of population declines, such as modification and degradation of limestone habitats through invasion of weed species and some farm management practices (e.g., grazing, herbicide use, over‐sowing and fertiliser application). As the majority of eastern South Island limestone occurs on private land (Heenan and Rogers 2019), securing parts of populations as formal conservation reserves or introducing greater legislative protection may be necessary.

We acknowledge that it is unrealistic to implement these measures for every population of *G. calcis*, however. Although other conservation genetic studies have also stressed the need to preserve many or all populations in plant species with high population structure (e.g., Silva et al. 2020; Wulff et al. 2017), insufficient resources available to undertake conservation management for all *G. calcis* MUs impose a challenging question: which populations or limestone sites should be conserved? Which should not be actively managed? In the first instance, a priority focus should be conserving one population of each of the subspecies. As these were shown to represent one of the highest levels of genetic structure in *G. calcis*, committing to this would preserve a large proportion of genetic diversity. If it is feasible to conserve a greater number of populations (e.g., multiple per subspecies), then other factors will contribute to decision‐making processes. These could include the degree of genetic distinctiveness (e.g., Xu et al. 2016), the presence of existing formal protection (e.g., Silva et al. 2020), census size and/or the presence of other threatened limestone species. The relative importance of these factors may be different for each subspecies. For example, in *G. calcis* subsp. *taiko*, either the larger Limestone Valley or the Totara Valley population could be prioritised over smaller populations as fewer resources will be needed to maintain their population at a size that facilitates long‐term persistence (Frankham et al. 2014). If additional resourcing is available, maximising genetic diversity for this subspecies could subsequently be prioritised by conserving a small, but genetically distinct population such as Mount Donald (< 200 plants; Table 1). In the case of *G. calcis* subsp. *calcis*, although both extant populations are similarly divergent relative to other *G. calcis* populations and have some formal protection (Miller 2023; Milliken et al. 2024), the Awahokomo population is larger than Earthquakes (which is also known as Waipata Scientific Reserve) (Table 1) and supports the highest concentration of narrow‐range limestone species in New Zealand (Rogers et al. 2018). The ongoing management of this location may therefore benefit other threatened taxa as well. If population size and the presence of other threatened limestone species are used as primary criteria for decision making, this population could be prioritised. For *G. calcis* subsp. *waipara*, it would be appropriate to prioritise conserving genetic diversity because of the small size of its extant populations and the high genetic divergence of this taxon from the other *G. calcis* subspecies. This would be best achieved by prioritising both the Mount Brown and Whiterock populations for conservation. However, as local conservation practitioners will have the greatest knowledge of limestone sites, they will be best placed to make management decisions strategically. By designating MUs, we provide a structure for this which will allow for informed management decisions that maximise the conservation of genetic diversity in *G. calcis* and assist in the conservation of vulnerable limestone ecosystems.

## Author Contributions

**Robb W. Eastman‐Densem:** conceptualization (equal), data curation (lead), formal analysis (lead), funding acquisition (equal), investigation (equal), methodology (lead), project administration (supporting), visualization (lead), writing – original draft (lead), writing – review and editing (lead). **David S. Glenny:** conceptualization (supporting), funding acquisition (supporting), methodology (supporting), supervision (supporting), writing – original draft (supporting), writing – review and editing (supporting). **Peter B. Heenan:** conceptualization (equal), funding acquisition (equal), methodology (equal), supervision (supporting), visualization (supporting), writing – original draft (supporting), writing – review and editing (supporting). **Jana R. Wold:** data curation (supporting), formal analysis (supporting), writing – original draft (supporting), writing – review and editing (supporting). **Pieter B. Pelser:** conceptualization (equal), data curation (supporting), formal analysis (supporting), funding acquisition (equal), investigation (equal), methodology (supporting), project administration (lead), supervision (lead), writing – original draft (equal), writing – review and editing (supporting).

## Ethics Statement

For all sampling on private land, landowner permission was obtained. Collections on public land utilised existing permits to Manaaki Whenua—Landcare Research.

## Conflicts of Interest

The authors declare no conflicts of interest.

## Supporting information


Data S1.

## Data Availability

All code used in data processing and analyses has been deposited on the University of Canterbury figshare https://doi.org/10.26021/canterburynz.27011971 and is also available on github: https://github.com/Robb‐ED/Gentianella‐calcis. As many landowners involved in this project wish for them and their properties to remain anonymous, raw reads and their associated metadata are stored on figshare under a permanent embargo https://doi.org/10.26021/canterburynz.27157051. Those wishing to access these data may contact the University of Canterbury library to be granted access.
