# Supplementary material for: Conservation Genomics for Threatened New Zealand Gentianella calcis (Gentianaceae) and Implications for Vulnerable Limestone Ecosystems
Source: Ecol Evol. 2025 Jun 17;15(6):e71596. doi: 10.1002/ece3.71596 (PMC12171645; doi:10.1002/ece3.71596)
Supplement: Supplementary file 1 — Data S1. [file ECE3-15-e71596-s001.docx]

Supplemental table 1: *Stacks* parameter combinations used in *Gentianella calcis* and *G. astonii* parameter trials with the corresponding number of *-R 80* loci (loci present in at least 80% of all samples) with and without replicate samples, and average SNP error used to inform parameter selection.

| *Stacks* Parameter Combination | Number of -*R* 80 RAD loci without replicates | Number of -*R* 80 RAD loci with replicates | Average SNP error |
| --- | --- | --- | --- |
| M1n1 | 3488 | 2227 | 0.0628 |
| M1n2 | 4229 | 2681 | 0.0658 |
| M2n1 | 3890 | 2495 | 0.0626 |
| M2n2 | 4553 | 2891 | 0.0623 |
| M2n3 | 4977 | 3222 | 0.0675 |
| M3n2 | 4967 | 3222 | 0.0698 |
| M3n3 | 5304 | 3446 | 0.0696 |
| M3n4 | 5610 | 3693 | 0.0752 |
| M4n3 | 5648 | 3691 | 0.0727 |
| M4n4 | 5848 | 3854 | 0.0737 |
| M4n5 | 6102 | 4014 | 0.0766 |
| M5n4 | 6045 | Not tested | |
| M5n5 | 6182 |  |  |
| M5n6 | 6381 |  |  |
| M6n5 | 6365 |  |  |
| M6n6 | 6463 |  |  |
| M6n7 | 6573 |  |  |

Supplemental table 2: Output of Analysis of Molecular Variance (AMOVA) as per the ‘poppr.amova’ in ade4 using dataset 2a containing *Gentianella calcis* and *Gentianella astonii* samples.

|  | DF | Sum Sq | Mean Sq |
| --- | --- | --- | --- |
| Among populations | 11 | 16001.217 | 1454.65606 |
| Within populations | 137 | 5317.418 | 38.81327 |
| Total | 148 | 21318.635 | 144.04483 |
| Components of covariance | | | |
|  | Sigma | % | |
| Variation among populations | 116.54110 | 75.0163 | |
|  |  |  | |
| Variation within populations | 38.81327 | 24.9837 | |
| Total variation | 155.35437 | 24.9837 | |

Supplementary table 3: Table of coefficients produced when using a linear regression to test for a relationship between sample size and heterozygosity.

|  | Estimate | Standard error | T value | Pr(>\|t\|) |
| --- | --- | --- | --- | --- |
| Intercept | 0.108487 | 0.033106 | 3.277 | 0.00833 |
| regression_data$Sample.Size | 0.002243 | 0.002397 | 0.936 | 0.37153 |

Supplementary table 4: Table of coefficients produced when using a linear regression to test for a relationship between sample size and F_IS_ (inbreeding coefficient).

|  | Estimate | Standard error | T value | Pr(>\|t\|) |
| --- | --- | --- | --- | --- |
| Intercept | -0.049533 | 0.028751 | -1.723 | 0.116 |
| regression_data$Sample.Size | -0.001843 | 0.002082 | -0.885 | 0.397 |


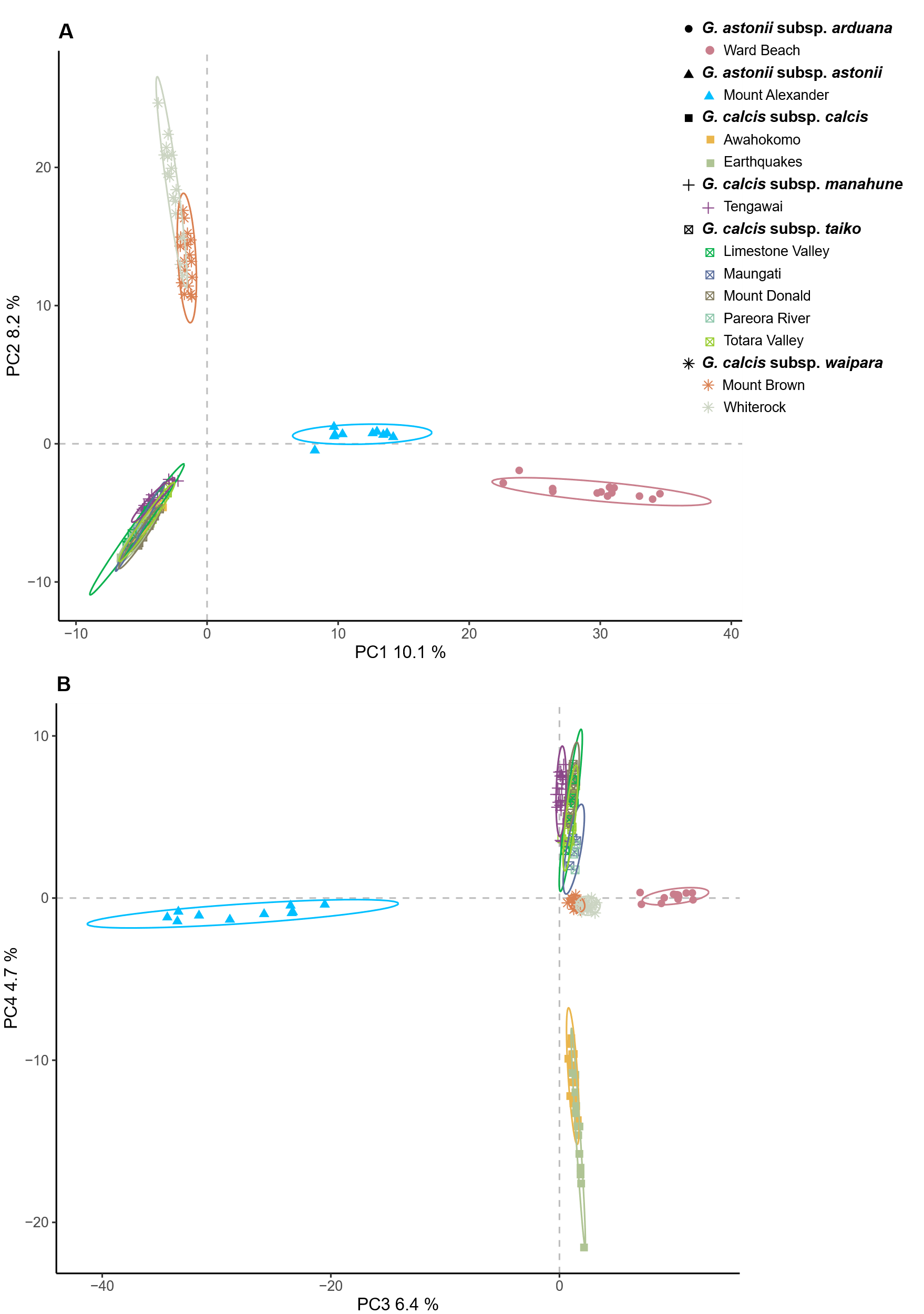


Supplemental figure 1: First two (A) and third and fourth axes (B) of PCA from *Gentianella calcis* and *G. astonii* SNPs using the same filtering procedure as for dataset 2a, except that a minimum minor allele count of 3 (--min-mac 3), was used as a filtering parameter in populations instead of a minimum minor allele frequency of 0.05.


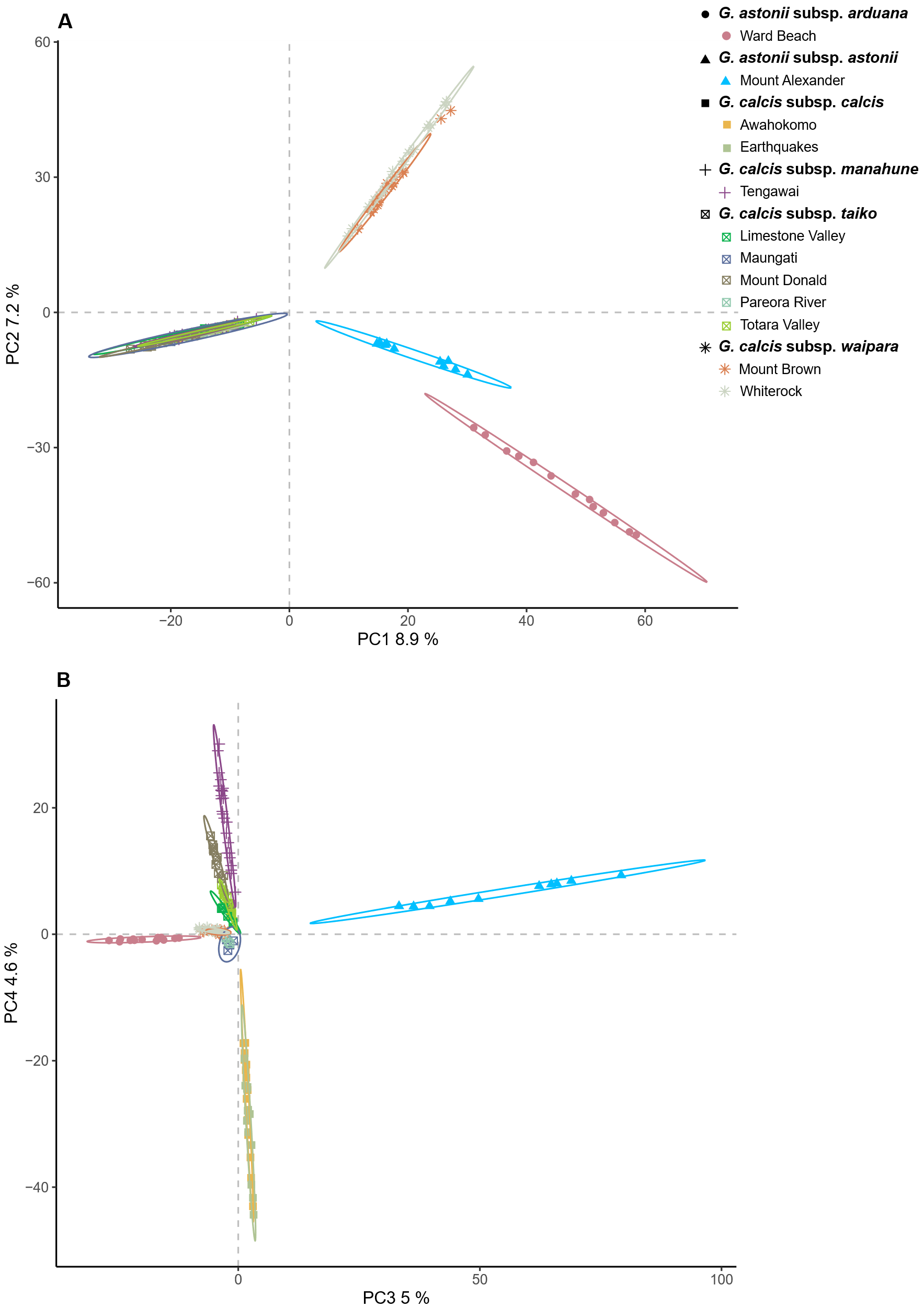


Supplemental figure 2: First two (A) and third and fourth axes (B) of PCA from *Gentianella calcis* and *G. astonii* SNPs using the same filtering procedure as dataset 2a, except that a minimum minor allele count of 3 (*--min-mac* 3), and RAD loci found in 60% of samples (*-R* 0.6) were used as filtering parameters in *populations* instead of a minimum minor allele frequency of 0.05 and *-R* 0.8 respectively.


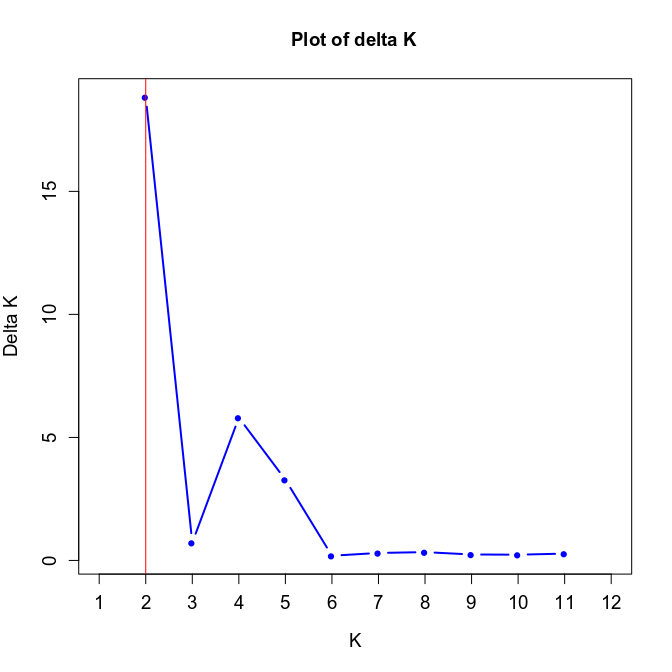


Supplemental figure 3: Plot of delta K values calculated by *Structure_Threader* for dataset 2a containing *Gentianella calcis* and *G. astonii* samples. The red line indicates the K value with the greatest associated delta K value.


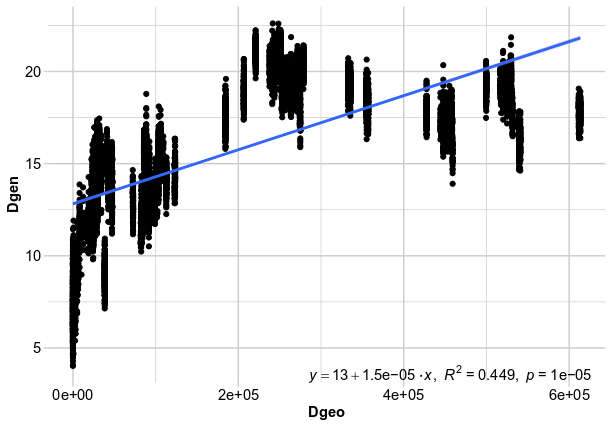


Supplemental figure 4: Output of the Mantel test as per gl.ibd from *dartR* used to test for a relationship in dataset 2a between Euclidean distance between sample coordinates (Dgeo) and Euclidean distance between *Gentianella calcis* and *Gentianella astonii* sample genotypes (Dgen).


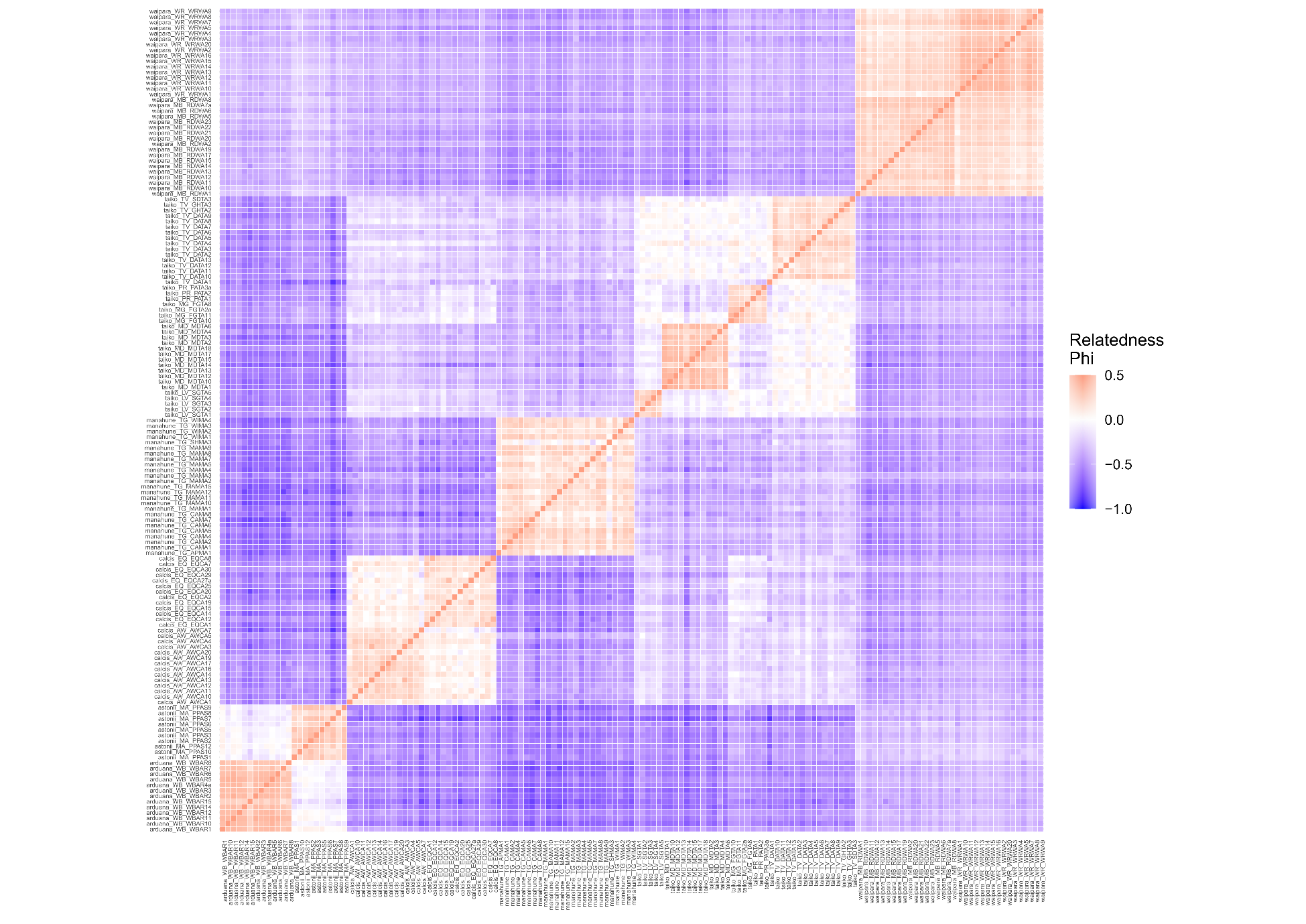


Supplemental figure 5: Heatmap of pairwise kinship coefficients (phi values) for each sample in dataset 2a produced by relatedness2 in *vcftools*. Samples are labelled by subspecies and abbreviated population name. Population abbreviations are as follows: WB: Ward Beach; MA: Mount Alexander; AW: Awahokomo; EQ: Earthquakes; TG: Tengawai; LV: Limestone Valley; MG: Maungati; MD: Mount Donald; PR: Pareora River; TV: Totara Valley; MB: Mount Brown; WR: Whiterock.
